# A Case of Herpes Simplex Virus Meningitis in an Immunocompromised Individual: Avoiding Common Diagnostic Pitfalls

**DOI:** 10.7759/cureus.42242

**Published:** 2023-07-21

**Authors:** Swechchha Silwal, Esraa Hassan, Shikha Jain, Ibtisam Rauf, Sri J Obulareddy, Sara Suleman, Faraaz A Yousuf, Eric O. Gomez Urena, Cristina Corsini Campioli, Nitesh K Jain

**Affiliations:** 1 Department of Internal Medicine, Trinity Health Oakland/Wayne State University, Pontiac, USA; 2 Department of Critical Care Medicine, Mayo Clinic Health System, Mankato, USA; 3 Department of Medicine, MV Jayaraman (MVJ) Medical College and Research Hospital, Bengaluru, IND; 4 Department of Medicine, St. George’s School of Medicine, University Centre Grenada, West Indies, GRD; 5 Department of Oncology/Hematology, University of Arkansas for Medical Sciences, Little Rock, USA; 6 Department of Palliative Care Medicine, Mayo Clinic Health System, Mankato, USA; 7 Department of Medicine, Rhodes College, Memphis, USA; 8 Department of Infectious Disease, Mayo Clinic Health System, Mankato, USA

**Keywords:** polymerase chain reaction (pcr), csf pleocytosis, amphetamine abuse, chronic alcohol abuse, alcohol abuse, immunocompromised status, lumbar puncture (lp), cerebrospinal fluid (csf), hsv-1, viral meningoencephalitis

## Abstract

Herpes simplex virus meningoencephalitis (HSV ME) is a severe viral infection that affects the brain and surrounding tissues. It is caused primarily by HSV type 1 (HSV-1) virus. This condition requires prompt recognition and treatment due to its potential for significant morbidity and mortality. We aim to highlight the importance of avoiding common diagnostic pitfalls in identifying HSV meningoencephalitis, especially in immunocompromised individuals. We present a case of a 34-year-old immunocompromised patient with HSV meningoencephalitis, emphasizing key clinical features and diagnostic strategies that helped us reach an accurate diagnosis. By sharing this case, we aim to enhance awareness and improve the management of HSV meningoencephalitis in similar patient populations, leading to better outcomes.

## Introduction

Herpes simplex virus (HSV) is one of the leading causes of meningitis and encephalitis, and the prognosis is impacted by timely diagnosis and initiation of treatment [1]. It can occur in all age groups, and about 90% of HSV encephalitis is due to HSV type 1 (HSV-1) [1]. HSV-1 spreads mainly through oral contact and is known to cause oral sores. Meningitis and encephalitis are usually from the reactivation of a virus from earlier infection [2]. Meningoencephalitis (ME) symptoms include fever, headache, neck stiffness, and confusion. In addition, patients may have hallucinations, gastrointestinal symptoms, and generalized rash [3]. However, immunocompromised individuals can present with atypical symptoms such as lack of systemic symptoms or prodrome, meningism, fewer focal motor deficits, less pronounced cerebrospinal fluid (CSF) white cell count pleocytosis, recurrent brain stem encephalitis, and more widespread involvement beyond temporal lobe lesions on computed tomography (CT) scan and magnetic resonance imaging (MRI) of the brain [1,4]. Diagnostic testing with polymerase chain reaction (PCR) of cerebrospinal fluid (CSF) can be positive, but leukocytic pleocytosis might not be evident in an immunocompromised patient. Due to this, HSV ME can be misdiagnosed and underreported [4]. Nevertheless, using a targeted PCR in addition to multiplex PCR has been shown to improve diagnostic yield [5]. Untreated HSV ME has a high mortality, over 70% [1]. Here, we present a case of HSV ME in a 34-year-old immunocompromised individual, illustrating avoidance of common diagnostic pitfalls.

## Case presentation

A 34-year-old male with a past medical history of anxiety disorder, alcohol abuse, and methamphetamine use disorder presented to the emergency room (ER) for evaluation of fever, headache, and neck pain. The symptoms started on the day of presentation after using methamphetamine. He was tachycardic but thought to be due to methamphetamine use, and he was discharged as his laboratory results were reassuring and symptoms improved after benzodiazepines were administrated. However, he returned shortly to the ER in four hours because of worsening headache, neck pain, and fever. On arrival, he was ill-looking and in discomfort. Vitals showed a heart rate of 152/minute and a respiratory rate of 30/minute. Laboratory investigations are shown in Table 1.

**Table 1:** Basic laboratory findings at admission AST: aspartate aminotransferase, ALT: alanine aminotransferase, ALP: alkaline phosphatase, BUN: blood urea nitrogen

Admission laboratory test and vitals	Value	Reference
Sodium	137 mmol/L	135-145 mmol/L
Potassium	3.7 mmol/L	3.6-5.2 mmol/L
Chloride	105 mmol/L	98-107 mmol/L
Bicarbonate	20 mmol/L	22-29 mmol/L
BUN	18 mg/dL	8-24 mg/dL
Creatinine	1.28 mg/dL	0.74-1.35 mg/dL
Total calcium	-	8.6-10 mg/dL
AST	55 U/L	8-48 U/L
ALT	51 U/L	7-55 U/L
ALP	84 U/L	40-129 U/L
Total bilirubin	1.2 mg/dL	≤1.2 mg/dL

**Figure 1:**
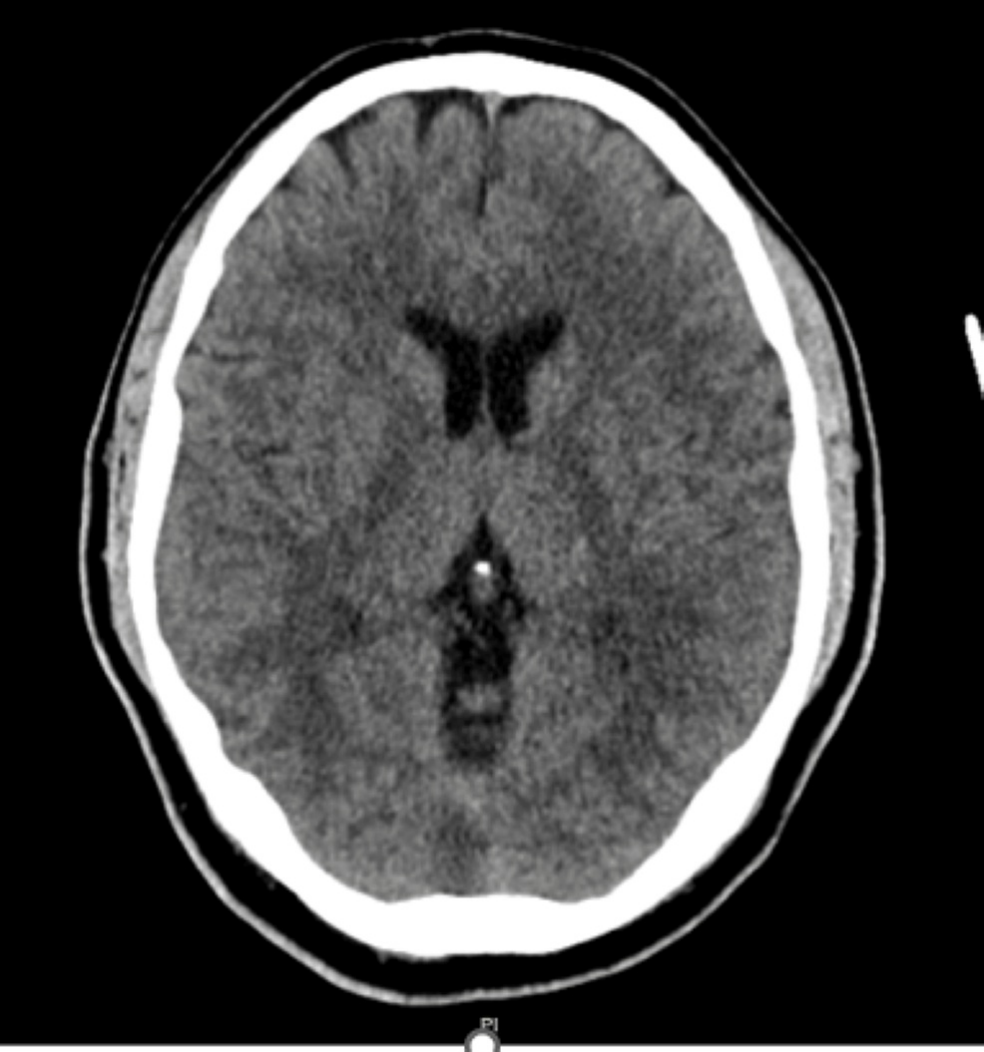
Computed tomography scan of the head without contrast: no acute abnormalities were noted

On examination, he was alert and oriented but had neck rigidity. There was no agitation or clonus, but on laboratory findings, we noted a fever of 38.7°C, leucopenia of 1.2 × 10^9^/L, and lactic acidosis of 4.1 mmol/L. Principal differential diagnoses included sepsis from central nervous system infection such as bacterial or viral meningitis/encephalitis and methamphetamine toxicity. The patient initially was alert and oriented with blood pressure (BP) of 108/58 mm Hg. During his emergency room (ER) stay, he became progressively encephalopathic with a decline in BP. He was given intravenous fluid as per sepsis protocol at 30 cc/kg along with broad-spectrum antibiotics, vancomycin, cefepime, and metronidazole. However, he remained hypotensive (83/53 mm Hg), encephalopathic, and difficult to arouse in the ER. Norepinephrine infusion was therefore initiated, and he was admitted to the intensive care unit (ICU). During this time, a comprehensive infectious workup was ordered, including a computed tomography (CT) scan of the head in the ER (Figure 1), an MRI of the spine with and without contrast, blood cultures, and a lumbar puncture (LP). Imaging studies demonstrated no significant abnormality, and cerebrospinal fluid (CSF) analysis showed erythrocytosis with normal white cell count (1/mcL) with 100% lymphocytes on differential (Table 2).

**Table 2:** CSF laboratory findings CSF: cerebrospinal fluid, RBC: red blood cells, WBC: white blood cells, PCR: polymerase chain reaction, HSV: herpes simplex virus

CSF laboratory test	Value	Reference
CSF appearance	Clear	Normal
RBC	7/mcL	<10/mcL
WBC	1/mcL	0-5/mcL
Lymphocyte	100%	60% +/- 20%
Protein	28 mg/dL	15-45 mg/dL
Glucose	66 mg/dL	Approximately 60% of plasma (plasma glucose = 111)
Xanthochromia	Negative	Negative
Gram stain	No organism seen	Negative
CSF culture	No growth after seven days of incubation	Negative
Meningitis/encephalitis panel, PCR (including HSV-1 and HSV-2)	Negative	Negative
Varicella-zoster virus PCR	Negative	Negative
HSV-1 PCR	Positive	Negative
HSV-2 PCR	Negative	Negative

Acyclovir was added to therapy to empirically cover viral meningitis in the ICU. His encephalopathy was initially considered secondary to the additional benzodiazepines he received while in the ER. During his ICU stay, targeted PCR for herpes simplex virus-1 on CSF came back positive, yielding the diagnosis of HSV-1 meningoencephalitis. Of note, the CSF meningitis/encephalitis panel for HSV-1 was negative. Blood cultures remained negative, and the patient was continued on a 10-day course of acyclovir. The patient remained afebrile, and hemodynamic stability was regained. He was discharged to a rehabilitation facility for drug detoxification.

## Discussion

HSV is one of the most common causes of viral encephalitis. Its incidence in the USA is one in 100,000-150,000 [6]. HSV-1 is more common to cause encephalitis than HSV-2 [6-8]. HSV meningoencephalitis (HSV ME) is associated with high mortality, morbidity, and financial burden; untreated mortality approaches 70%, and among those who survive, the vast majority have some degree of neuropsychiatric impairment [8]. Hence, it is important to be aware of some common pitfalls relating to diagnosis.

Patients with HSV ME may present atypically, and consequently, the diagnosis may be missed if appropriate tests, such as MRI of the brain and CSF PCR studies, are not ordered [8]. Classically, patients present with viral prodromal symptoms, altered mental sensorium, fever, seizures, focal neurological deficits, and behavioral changes, accompanied by abnormal electroencephalogram (EEG), gadolinium contrast enhancing changes on MRI of the brain, CSF pleocytosis (>5 nucleated cells/mL), and CSF PCR testing positive if tested at the appropriate time [9,10]. However, it is important to have a high index of suspicion as certain patients, especially those who are immunocompromised, may not present with the typical constellation of signs or symptoms [8,11,12]. In one study, immunocompromised patients with HSV ME have been noted to have viral prodromal symptoms and focal signs and symptoms infrequently [13]. Lack of CSF pleocytosis and more severe neurological involvement were reported outside the meso-temporal regions in imaging studies [13].

It is also well-recognized that there could be a lack of CSF pleocytosis in the immunocompromised population, which was also noted in our case [8,10]. This lack of CSF pleocytosis is common in immunocompromised patients with bacterial meningitis and HSV ME, alluding to a defect in immune defense mechanisms [8,11,12]. Hence, it is essential to perform pertinent diagnostic studies such as brain MRI and targeted CSF PCR for HSV, which have high diagnostic values when performed with a high index of suspicion [8]. At the same time, CSF PCR for HSV, when performed too early in the disease course, could be negative [8,14]. In such cases, it is recommended that one should treat the disease and repeat the testing about three to seven days later when it may test positive [14]. This is clinically relevant as morbidity and mortality were almost six times higher in immunocompromised patients when compared to immunocompetent patients [8,13]. Of note, multiplex molecular panels may demonstrate lower analytic sensitivity compared to dedicated targeted singleplex PCR assays; hence, if clinically relevant, the concomitant use of both molecular testing is recommended, as was done in our case, and only the targeted CSF HSV-1 PCR test was positive [5,15]. Nevertheless, as molecular testing sensitivity increases, so does the chance of a false-positive or true-positive result that is not clinically significant [15].

Our patient had a history of alcohol and methamphetamine drug abuse, thus qualifying himself for immunocompromised status [16-18]. He presented with fever, headache and tachycardia, and leukopenia. Initially, he was discharged back home as suspicion of a severe pathology was low. Fortunately, he presented again with more severe signs and symptoms, which led to more invasive testing such as lumbar puncture and CT of the head. The CSF studies demonstrated a lack of pleocytosis, but we continued with antimicrobial therapy until the targeted CSF HSV PCR returned positive, as our suspicion for HSV ME was high. The antibacterial agents were discontinued at that point, and a course of intravenous acyclovir was completed. The patient has been noted to be doing well and undergoing drug detoxification therapy and rehabilitation as an outpatient three months post-discharge.

## Conclusions

In immunocompromised patients, it is important to maintain a high index of suspicion for HSV meningoencephalitis and other infectious central nervous system pathologies as the clinical presentation may be subtle with an absence of typical signs and symptoms. Performing appropriate testing, such as timely MRI of the brain and CSF HSV PCR testing, in such a setting may unmask the diagnosis, preventing catastrophic outcomes.
